# Untreated vs. Treated Carbon Felt Anodes: Impacts on Power Generation in Microbial Fuel Cells

**DOI:** 10.3390/mi14122142

**Published:** 2023-11-23

**Authors:** Abdelghani Ghanam, Sebastien Cecillon, Andrei Sabac, Hasna Mohammadi, Aziz Amine, François Buret, Naoufel Haddour

**Affiliations:** 1 Univ Lyon, Ecole Centrale de Lyon, INSA Lyon, Université Claude Bernard Lyon 1, CNRS, Ampère, UMR5005, 69130 Ecully, Francefrancois.buret@ec-lyon.fr (F.B.); 2 Chemical Analysis and Biosensors Group, Laboratory of Process Engineering and Environment, Faculty of Science and Techniques, Hassan II University of Casablanca, B.P 146, Mohammedia 20000, Moroccoa.amine@univh2m.ac.ma (A.A.)

**Keywords:** carbon felt (CF), microbial fuel cell, anode materials, biofilm, power density, electroactive bacteria

## Abstract

This research sought to enhance the efficiency and biocompatibility of anodes in bioelectrochemical systems (BESs) such as microbial fuel cells (MFCs), with an aim toward large-scale, real-world applications. The study focused on the effects of acid-heat treatment and chemical modification of three-dimensional porous pristine carbon felt (CF) on power generation. Different treatments were applied to the pristine CF, including coating with carbon nanofibers (CNFs) dispersed using dodecylbenzene sulfonate (SDBS) surfactant and biopolymer chitosan (CS). These processes were expected to improve the hydrophilicity, reduce the internal resistance, and increase the electrochemically active surface area of CF anodes. A high-resolution scanning electron microscopy (HR-SEM) analysis confirmed successful CNF coating. An electrochemical analysis showed improved conductivity and charge transfer toward [Fe(CN)6]^3−/4−^ redox probe with treated anodes. When used in an air cathode single-chamber MFC system, the untreated CF facilitated quicker electroactive biofilm growth and reached a maximum power output density of 3.4 W m^−2^, with an open-circuit potential of 550 mV. Despite a reduction in charge transfer resistance (R_ct_) with the treated CF anodes, the power densities remained unchanged. These results suggest that untreated CF anodes could be most promising for enhancing power output in BESs, offering a cost-effective solution for large-scale MFC applications.

## 1. Introduction

Microbial fuel cells (MFCs) have emerged as a promising technology for the direct conversion of chemical energy from organic substrates into electricity, leveraging the catalytic activity of electrochemically active bacteria (EAB) at the anode [1,2,3,4]. The electrons released during substrate oxidation at the anode are transferred to the cathode, typically via an oxygen reduction reaction. Despite the potential of MFCs for wastewater treatment and power generation, their real-world application remains limited due to challenges such as low power densities [5,6]. One of the primary factors influencing MFC performance is the efficiency of extracellular electron transfer (EET) at the anode, which is determined by the properties of the anode material [7]. Among the myriad of materials explored, carbon felt (CF) has emerged as a frontrunner. Its inherent biocompatibility, electrical conductivity, and 3D porous structure make it an ideal candidate for facilitating efficient EET in BESs [8,9,10,11]. However, while CF is a frontrunner among multiple materials explored, there is a growing consensus that its performance can be further enhanced through strategic functionalization and modification [8,10,11]. Recent studies have delved into the acid-heat treatment of CF to optimize its properties for MFC applications. Such treatments have been shown to modify the surface structure and chemistry of the carbon felt, leading to enhanced bioelectricity generation. For instance, a study by Simeon et al. highlighted the combined effects of electrode material, spacing, and substrate feeding frequency on MFC performance [12]. Another study by Miran et al. emphasized the role of iron oxide-modified carbon electrodes in tandem with sulfate-reducing bacteria for efficient bio-electricity generation [13]. Fatima and colleagues integrated a novel lignin-based carbon fiber felt bioanode in an MFC, demonstrating its potential for treating recalcitrant textile wastewater [14]. Additionally, Kim et al. employed microwave and sulfuric acid treatments on graphite granules, resulting in a significant boost in bioelectricity generation in MFCs [15]. Furthermore, recent advancements in electrode functionalization have highlighted the potential of carbon nanofibers (CNFs) to enhance the electrochemical and catalytic properties of electrodes [16,17]. For instance, the use of Mo-doped carbon nanofibers (Mo-CNFs) as an anode in MFCs, as demonstrated by Wu et al., resulted in a power density that was double that of electrodes with unmodified CNFs [18]. Such advancements highlighted the pivotal role of CNF-functionalized electrodes in optimizing MFC performance. The dispersion of CNFs using surfactants like dodecylbenzene sulfonate (SDBS) and biopolymers such as chitosan (CS) has been shown to further enhance the hydrophilicity, biocompatibility, and electrochemical properties of the anode, paving the way for more efficient and robust MFC systems [19,20,21]. Such functionalization techniques have been shown to promote a better dispersion of CNFs on the electrode surface, leading to improved electrochemical activity and stability [22]. The use of SDBS as an anionic surfactant has been particularly effective in achieving stable aqueous dispersions of CNFs, with the π-π stacking induced by the benzene ring in SDBS playing an important role in this stabilization [23]. Furthermore, the functionalization of electrode surfaces using CNFs dispersed with CS biopolymer has been identified as a promising strategy to augment their electrochemical and catalytic attributes. For example, Liu et al. (2018) presented a CNFs/CS nanocomposite as a biocompatible electrode material, which enhanced the electricity generation in microbial fuel cells [24]. Similarly, Plekhanova et al. proposed an innovative technique for the immobilization of bacterial cells on graphite anode surfaces using CNFs combined with CS [25]. This modification led to a notable improvement in MFC performance. While these modification processes can enhance performance, they also elevate the cost of electrodes, potentially hindering the mass production of anodes. As a result, the feasibility of implementing large-scale MFC technology with nanomaterial-modified anodes is still being explored. A recent study by Fonseca et al. highlighted that an MFC equipped solely with pristine CF achieved a maximum power density (P_max_) of 1.46 W m^−2^, surpassing many results from studies using modified anodes [8]. This suggests that utilizing unmodified electrodes could be beneficial for the widespread, real-world application of MFCs.

In light of these considerations, this study undertakes a thorough examination of the performance dynamics of pristine CF anodes in MFCs in comparison to their modified counterparts. Through a systematic evaluation of various treatments and modifications, this work seeks to glean insights into the ideal anode material for MFCs. The ultimate objective is to harmonize performance enhancement with cost-effectiveness, potentially facilitating the wider adoption of MFC technology across various applications [26,27]. In this investigation, multiple treatments were administered to assess their influence on the performance of CF-based anodes in MFCs. The anodes were subjected to six distinct conditions, encompassing both pristine CF (CF) and acid-heat-treated CF (A-CF) substrates. These were subsequently immersed in two separate aqueous dispersions of CNFs, dispersed using either the SDBS surfactant (CF@CNF-SDBS, A-CF@CNF-SDBS) or the CS biopolymer (CF@CNF-CS, A-CF@CNF-CS). High-resolution scanning electron microscopy (HR-SEM) was employed to analyze the surface morphology of all anode materials. Electron transfer dynamics across these anode surfaces were probed using cyclic voltammetry (CV) and electrochemical impedance spectroscopy (EIS). The electrochemical characterization and performance of MFCs, equipped with different anodes, were examined and compared using single-chamber bottle-type MFC in batch mode. The electrochemical behavior and extracellular electron transfer were assessed using CV in MFC reactors at biofilm maturation.

## 2. Materials and Methods

### 2.1. Chemicals and Materials

Sulfuric acid (H_2_SO_4_, 95.0–98.0%), sodium dodecylbenzene sulfonate (SDBS), chitosan (CS), carbon nanofibers (CNF, pyrolytically stripped, platelets (conical), >98% carbon basis, D × L 100 nm × 20–200 µm), agarose, KCl, Nafion (5 wt.%), platinum on carbon black (10 wt.% Pt/C), sodium acetate (NaAc), and 2-propanol (≥99.5%) were sourced from Sigma-Aldrich (France) and utilized without further purification. A polytetrafluoroethylene (PTFE) spray solution (3 in 1) was procured from Castorama (Dardilly, France). All other reagents employed in this study were of analytical grade. Primary wastewater effluent (7 mS cm^−2^), serving as an electrolyte, and anaerobic activated sludge, used as a source of electrochemically active bacteria (EAB) in a NaAc (10 mM) medium, were collected from the Grand Lyon domestic wastewater treatment plant (Lyon, France). These were utilized for the initiation of single-chamber bottle-type microbial fuel cells (Figure S1) operated in batch mode.

### 2.2. Pretreatment and Modification of Carbon Felt-Based Anodes

**Figure 1:**
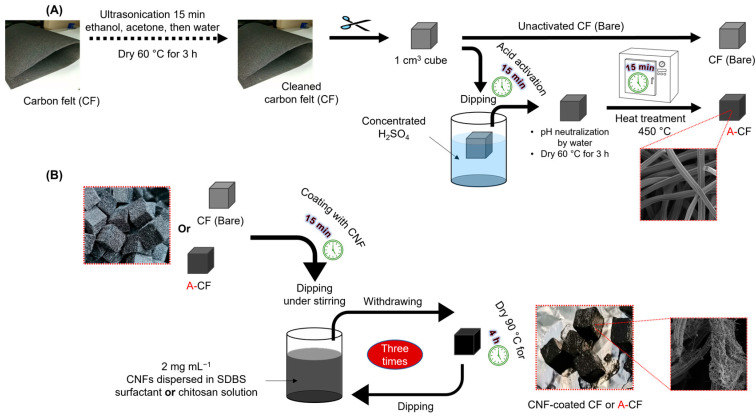
(**A**) Schematic of the preparation of CF and activated CF (A-CF) anode substrates and their dipping (**B**) for three cycles in a conductive CNF dispersion solution using SDBS surfactant or CS as dispersing agents.

As depicted in Figure 1, a series of six anodes were fabricated through acid activation, heat treatment, and immersion in CNF dispersions of CF anodes. The anode substrates, both pristine CF and A-CF, were individually immersed in two CNF dispersions utilizing SDBS surfactant and CS as dispersing agents. Initially, the pristine CF underwent sequential pretreatment with three distinct solvents (ethanol, acetone, and water), each subjected to 15 min of ultrasonication (Figure 1A). Following a drying period at 60 °C for 3 h, the CF was sectioned into 1 × 1 × 1 cm^3^ cubes and immersed in concentrated H_2_SO_4_ for 15 min under agitation. After multiple water rinses to achieve a neutral pH, the CF anodes were dried at 60 °C for another 3 h. The acid-treated CF electrodes then underwent a heat treatment at 450 °C for 15 min in a muffle furnace to remove manufacturing impurities [19,28]. This acid and heat treatment process aimed to diminish the internal resistance of CF, enhance hydrophilicity, and foster the creation of functional group-rich CF surfaces, potentially promoting superior adhesion of electroactive biofilms [20]. To further optimize the internal resistance and conductivity of both pristine (CF) and activated CF (A-CF), a chemical modification was undertaken (Figure 1B). This modification, designed to enhance interactions between electrogenic bacteria and the anode surface, involved the preparation of electrically conductive CNF dispersions. CF and A-CF were coated with CNF through a straightforward immersion and drying procedure. Aqueous CNF dispersions (2 mg mL^−1^) were prepared by ultrasonically treating CNFs in two distinct solutions of dispersing agents (SDBS surfactant and CS biopolymer) for 2 h. Both SDBS and CS solutions were prepared at concentrations of 2 mg mL^−1^ in water and 1% (*v*/*v*) acetic acid, respectively. These agents ensured stable and efficient CNF dispersion. The immersion and drying steps were repeated thrice to augment CNF loading and reduce the internal resistance of either pristine CF or A-CF. In the final step, all fabricated anodes were connected to the external circuit using stainless steel wire. Before MFC assembling and operations, the electrochemical activities of the prepared CF anodes were evaluated using CV and EIS techniques in 0.1 M KCl solution containing 10 mM [Fe(CN)_6_]^3−/4−^ redox probe. This is to evaluate and characterize the electron transfer at the surface of the synthesized anode materials. The redox probe and KCl were prepared in ultrapure water (18.2 MΩ cm^−2^). These anodes were then ready for characterization and deployment in MFC reactors.

### 2.3. Fabrication of Reference Electrodes

Due to the limitations of commercial Ag/AgCl reference electrodes (REs) in terms of size, a cost-effective, miniaturized Ag/AgCl (saturated KCl) RE was fabricated. The fabrication process was executed in three distinct phases. (i) Ag/AgCl formation: Silver wires were partially immersed in a 50 mM FeCl_3_·6H_2_O solution for a duration of 1 min (Figure S2). This immersion facilitated the formation of an AgCl film on the silver wires, in accordance with the specified redox reactions [28]. Subsequently, the silver wires, now coated with the AgCl film, were transferred to a saturated KCl solution. (ii) Preparation of ion-conducting agarose hydrogel: This phase involved the creation of a conductive hydrogel to act as a salt bridge at one end of a Pasteur pipette. The hydrogel was formulated by dispersing 1% (*w*/*v*) agarose in a saturated KCl solution (0.2 g in 20 mL). This mixture was subjected to microwave irradiation for a total of 1 min (in two 30-s intervals) to ensure complete agarose dispersion. The resultant dispersion was then placed on a preheated hotplate set at 80 °C and stirred vigorously. Concurrently, aliquots of the solubilized agarose solution were injected into one end of Pasteur pipettes and subsequently cooled in a saturated KCl solution. (iii) Assembly of the reference electrode: Once the hydrogel solidified, the pipettes were filled with saturated KCl. The previously prepared Ag/AgCl electrodes were then inserted, yielding Ag/AgCl (saturated KCl) REs analogous to commercial variants (Figure S3). The opposite ends of the Pasteur pipettes were sealed using silicone and left to dry at room temperature prior to their deployment in MFC systems. The potential of these home-made reference electrodes was measured against commercial AgCl/Ag reference electrodes, and only those electrodes exhibiting a shift of less than 10 mV were retained. This potential difference was noted for each electrode and considered to adjust the potential values presented.

### 2.4. MFCs Configuration, Inoculation, and Operation

**Figure 2:**
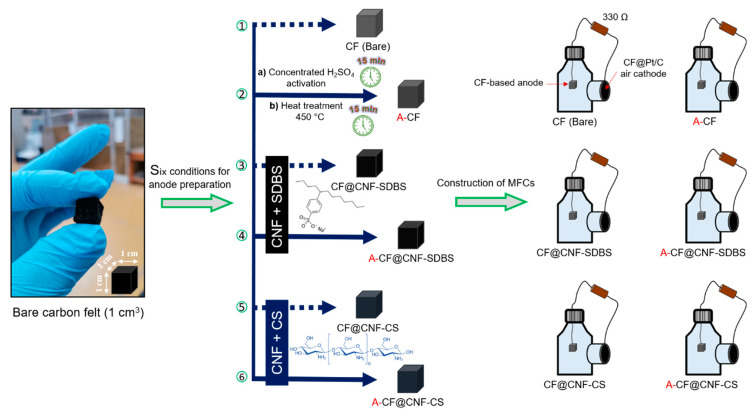
A detailed scheme summarizing the sequential step preparation of CF anodes and their utilization in single-chamber bottle-type MFCs. Both pristine CF and CF directly treated with concentrated H_2_SO_4_ and thermally involved at 450 °C (A-CF) were used as anode substrates for CNF coating. The prepared CNF dispersions (CNF in SDBS or CS) served as immersion solutions for dipping CF and A-CF.

Single-chamber batch MFCs were set up using 250 mL Wheaton bottles in the laboratory at ambient temperature. The cathode was prepared with a PTFE coating and a 5% platinum catalyst, following the procedure outlined by Cheng et al. [21]. The fabricated electrodes, encompassing cathodes, anodes, and reference electrodes, were integrated into single-chamber MFCs, as depicted in Figure 2. Each MFC system incorporated an anode material in the form of a 1 × 1 × 1 cm^3^ CF substrate cube. The MFC chambers were filled with 250 mL of primary effluent (7 mS cm^−2^), supplemented with 5 g L^−1^ of activated anaerobic sludge sourced from the Grand Lyon municipal wastewater treatment plant (Lyon, France), and enriched with NaAc (1 g L^−1^) medium to serve as a carbon substrate. Throughout the MFC operation, the cathode and anode in each reactor were interconnected through an external circuit, bridged by a 330 Ω resistor. This setup facilitated the formation of an electroactive biofilm on the anode surface and enabled electron transfer from the anode to the cathode. The spatial separation between the anode and cathode was maintained at approximately 2 cm. Additionally, all single-chamber MFCs were interfaced with data acquisition systems to continuously monitor and record voltage output during the maturation of the anodic biofilm every 1 min with a precision of 1 µV (Figure S4).

### 2.5. Electrochemical and SEM Characterization

The electrochemical characterization of the anode surfaces was achieved at ambient temperature using CV and EIS techniques within a conventional three-electrode electrochemical cell system, interfaced with a potentiostat (OGS 500, Origalys, Rilleux-La-Pape, France). These electrochemical measurements were designed to assess electron transfer mechanisms. As depicted in Figure S5, the three-electrode assembly consisted of uncoated or CNF-coated CF electrodes as working electrodes (WE), a stainless steel rod as a counter electrode (CE), and a commercially available Ag/AgCl electrode as a reference electrode (RE). The electrolyte used was a 0.1 M KCl solution containing a 10 mM [Fe(CN)_6_]^3−/4−^ redox probe. The CV measurements were conducted in the fixed potential range of −1 V to +1 V (vs. Ag/AgCl) at a scan rate of 100 mV s^−1^. EIS was carried out in the frequency range of 100 kHz to 10 mHz at the open circuit potential (OCP) with 10 mV s^−1^ amplitude. Upon stabilization of the voltage output (indicative of biofilm maturation) as recorded by data logging systems, the external circuit was opened and then reconnected with a potentiostat in a two-electrode arrangement. Then, each anode electrode of the MFCs was connected as WE, while the air cathode was connected to the potentiostat’s two shorted RE and CE cables. Afterward, the polarization curves (V-I) of the six different anodes were recorded using linear seep voltammetry (LSV) over a potential range from OCP values to 0 V at a scan rate of 10 mV s^−1^. Moreover, the power density (P, W m^−2^) curves were obtained by multiplying the cell voltage by current density (J, A m^−2^) according to Equation (1).

(1) P=V×I/S

where V represents the measured voltage and *I* denotes the current normalized to the projected anode surface area (*S*). On the other hand, the anodic biofilm formed at the six anodes was characterized using CV, which might provide information including the electrochemical activity and electroactive species involved in charge transfer. Indeed, the electroactivity of anodic biofilms or the load in electrochemically active bacteria (EAB) could be assessed as a function of the current intensities related to its typical redox peaks in CV [29]. The CV curves at biofilm maturation were recorded in the potential range of −800 mV to 700 mV (vs. Ag/AgCl) at a scan rate of 10 mV s^−1^ in MFC reactors containing wastewater and 10 mM NaAc substrate. Furthermore, the surface morphology of all prepared anode materials based on CF substrates was investigated using field emission scanning electron microscopy (SEM) (SEM TESCAN model MIRA-3 (TESCAN-ORSAY, Brno, Czech Republic)).

## 3. Results and Discussion

### 3.1. Surface Morphology of CF Electrodes

**Figure 3:**
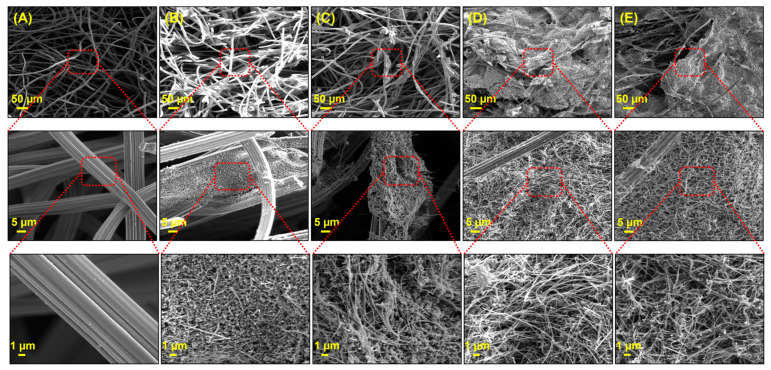
SEM images of (**A**) pristine CF, (**B**) CNF/CS-coated CF, (**C**) CNF/CS-coated A-CF, (**D**) CNF/SDBS-coated CF, and (**E**) CNF/SDBS-coated A-CF anodes.

After the successful preparation of uncoated and CNF-coated CF anodes, an SEM analysis was performed to examine the surface morphology before and after modification of all prepared anodes, as shown in Figure 3. As can be seen, the pristine CF anode was made up of carbon fibers with a diameter of approximately 10 µm, which networked and formed a 3D macroporous structure. Furthermore, the carbon fibers of the pristine CF have a smooth surface. Figure 3B and Figure 3C demonstrate that the pristine CF and A-CF were successfully coated with CNF dispersed in a CS solution (i.e., the carbon fibers were covered by the CNF-CS composite), respectively. Similarly, CNF dispersed in an SDBS surfactant solution coated the total volume of both CF and A-CF anode substrates, as shown in Figure 3D and Figure 3E, respectively. The CNF was embedded in the 3D macroporous structure. A notable increase in the roughness of individual carbon fibers is evident post CNF coating, suggesting a potential enhancement in specific surface area without compromising the structural integrity of the fibers. As observed in Figure 3B,C, the CNF-CS particles tend to agglomerate, resulting in the formation of grain-like structures. In contrast, the CNF-SDBS composites, as seen in Figure 3D,E, manifest a distinct mesh-like network within a 3D microstructure. These results provided a detailed insight into the surface morphology of both uncoated and CNF-coated CF anodes. The distinct structural differences between the pristine CF and the CNF-coated versions, whether dispersed in CS or SDBS, underscore the potential for tailored surface properties. Such modifications, especially the increased roughness and specific surface area, could play a main role in enhancing the electrochemical performance of the electrodes. To elucidate the rationale behind the distinct microstructures observed in Figure 3, it is essential to understand the interaction dynamics between the coating materials and the CF substrate. The CNF-CS composite leads to grain-like structures due to the adhesive and film-forming properties of chitosan. This results in a more pronounced agglomeration of CNF particles, enhancing the surface roughness and potentially the bioactivity of the anode. In contrast, the CNF-SDBS dispersion creates a mesh-like network within the CF’s 3D microstructure. The surfactant properties of SDBS facilitate a more uniform CNF dispersion, preventing particle aggregation and ensuring a more intricate and evenly distributed coating. This microstructure variation is significant for electrochemical applications, as it directly impacts the surface area and the electrode’s biofilm-interaction capabilities. These observations underline the importance of coating material selection in tailoring anode surface properties for specific applications in MFCs. The distinct microstructural features achieved through different CNF coatings demonstrate the potential for enhancing electrode performance through careful material engineering.

### 3.2. Electrochemical Properties of Unmodified and Modified CF Electrodes

**Figure 4:**
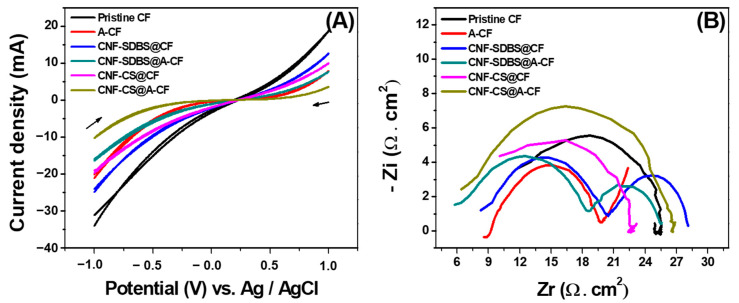
(**A**) CVs at different CF-based anode surfaces of 10 mM [Fe(CN)_6_]^3−/4−^ and 0.1 M KCl in the potential range of −1 to 1 V vs. Ag/AgCl. Scan rate 100 mV s^−1^. (**B**) Corresponding Nyquist plots at OCP values.

The electron transfer capabilities of all anode surfaces were evaluated using CV and EIS techniques within a conventional three-electrode electrochemical cell system, utilizing 10 mM [Fe(CN)_6_]³^−^/⁴^−^ as the redox probe. As depicted in Figure 4A, the CV profiles, spanning a potential range of −1 V to +1 V vs. Ag/AgCl at a scan rate of 100 mV s^−1^, did not exhibit pronounced redox peaks. However, the chemical and thermal treatments, as well as the subsequent CNF modification of the pristine CF anode, expanded the potential window and reduced the resistive current. This observation was further corroborated by EIS measurements conducted at OCP, which indicated a reduction in charge transfer resistance (R_ct_) post chemical and thermal treatments or modifications (Figure 4B). The expanded potential window and reduced resistive current observed post treatments suggest an increase in active sites for electron transfer. Notably, the Nyquist plots for CNF-coated CF/A-CF, where CNF was dispersed using the SDBS agent, presented two distinct semicircles: a prominent one at high frequencies followed by a smaller one at low frequencies. This dual-semicircle behavior is indicative of the altered electrochemical dynamics due to the SDBS-assisted CNF distribution on the CF/A-CF anodes. These findings underscore the role of chemical and thermal treatments, as well as CNF modifications, in enhancing the electron transfer capabilities of CF electrodes. The distinct electrochemical behavior observed with SDBS-dispersed CNF coatings highlights the potential for tailored surface interactions, crucial for optimizing MFC performance.

### 3.3. Performance of the MFC Equipped with Various Anode Materials

#### 3.3.1. Biofilm Growth on Various CF-Based Anode Surfaces

**Figure 5:**
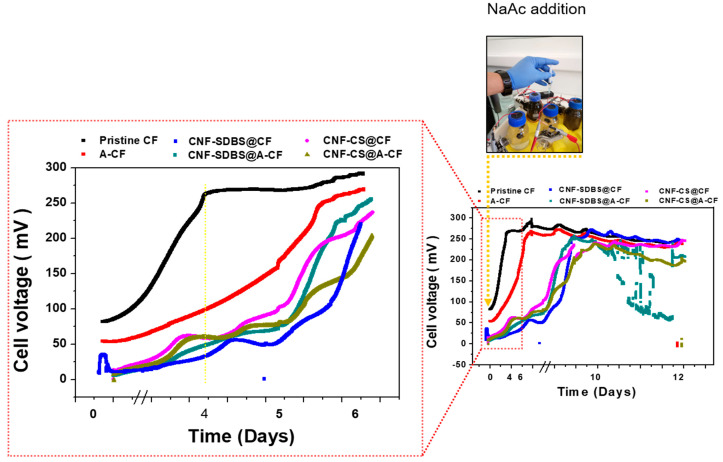
The voltage outputs produced in MFCs equipped with different anode materials. The anode and cathode in each MFC system were connected via an external circuit across a 330 Ω resistor.

As shown in Figure 5, the voltage outputs of the MFCs were recorded over an 11-day period to monitor biofilm growth on the six CF anodes, which included uncoated and CNF-coated pristine CF or A-CF anodes. As observed, the voltage of the MFC equipped with a pristine CF anode increased more rapidly, reaching 275 mV during the first four days of MFC running, and then slightly increased to reach a nearly stable value of 300 mV. Although the acid-heat treatment could increase the electrical conductivity and hydrophilicity of the CF [30], the biofilm growth kinetics were still lower than those of pristine CF. These results suggest that electrogenic bacteria are more likely to adhere/colonize faster on hydrophobic surface [31]. The acid-heat treatment or coating the CF/A-CF electrodes with CNF increased their conductivity and enhanced charge transfer rate. However, in the MFC configuration, pristine CF demonstrated the best performance in terms of its high affinity towards electroactive bacteria and faster biofilm formation. In addition, the results obtained are consistent with those reported in a recently published study, which showed that chemical treatments did not bring much improvement in power output [8]. Hence, it can be concluded that pristine CF alone may be sufficient to achieve satisfactory electrical performance in MFC systems.

#### 3.3.2. Electrochemical Activity of the Developed Biofilms

**Figure 6:**
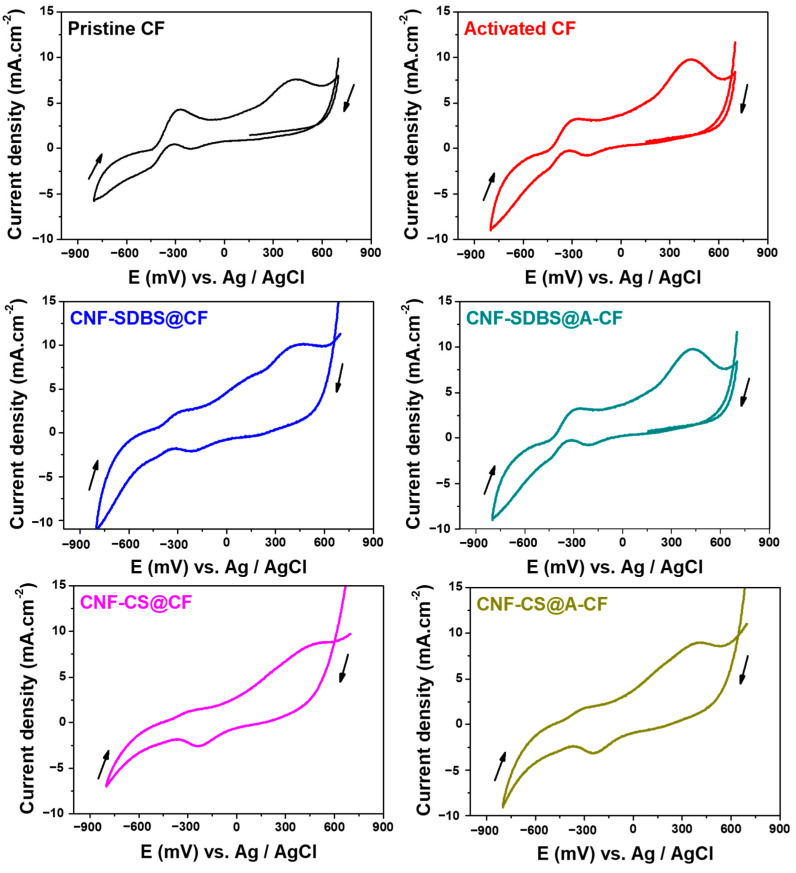
CV curves depicting the electrochemical behavior of biofilms developed on different anode surfaces after 11 days (biofilm maturation) running MFC systems containing wastewater with 10 mM NaAc substrate. Scan rate 10 mV s^−1^.

As shown in Figure 6, the electrochemical behavior of the biofilm developed at different anode materials was investigated using CV in MFC reactors. This is to assess extracellular electron transfer (EET) between the anodic biofilm and the electrode surface through the bioelectrocatalytic oxidation of the NaAc substrate. Indeed, after 11 days of MFC operation, all CVs exhibited a reversible redox system at around −300 mV vs. Ag/AgCl, with an oxidative peak at 400 mV, indicating that direct electron transfer was the primary EET mechanism, as previously described [32]. Based on values in the literature, the negative potential region is typically associated with mediated electron transfer in Shewanella oneidensis and/or heterogeneous electron transfer via nanowires characteristic of *Geobacter sulfurreducens* EAB [33,34]. In the positive potential domain, direct electron transfer predominates as the primary EET mechanism, potentially facilitated by c-type cytochromes, as observed in genera such as *Clostridium*, *Geobacter*, and *Shewanella* [35]. The congruence in CV profiles across all MFC anodes indicates that the electron transfer dynamics within the biofilms are comparable between untreated and treated CF anodes. However, the pristine CF anode displayed the most pronounced peak intensity with distinct redox peaks in the negative potential region, while manifesting one of the lowest peak intensities in the positive potential region, relative to other CF anodes. As illustrated in Figure 5, the voltage output profiles in MFCs aligned with the observed peak intensities in the negative potential region. This suggests that the electron transfer mechanisms associated with this potential range play a pivotal role in determining MFC performance. A synergistic approach employing both metaproteomics and metagenomics would provide a more holistic understanding.

#### 3.3.3. Polarization Curves at Biofilm Maturation Conditions for Power Generation

**Figure 7:**
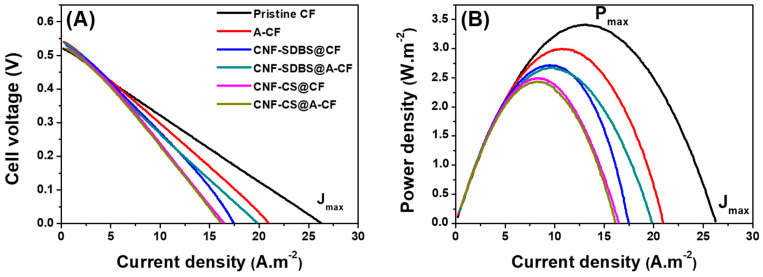
(**A**) Polarization curves recorded using LSV after 11 days running (biofilm maturation) of single-chamber MFCs equipped with different CF-based anodes and containing wastewater and 10 mM NaAc substrate, and (**B**) corresponding power density curves. Scan rate 10 mV s^−1^.

The performance of MFCs, gauged by maximum power and current densities utilizing both uncoated and CNF-coated carbon felt anodes, was rigorously examined. Upon stabilization of MFC voltage outputs, polarization and power density curves for the six distinct anodes were derived using LSV at a scan rate of 10 mV s^−1^, with each MFC interfaced in a two-electrode configuration to the potentiostat. Notably, the MFC equipped with a pristine CF anode produced maximum power density output and short-circuit current density, as illustrated in Figure 7 and Table 1. These results are also consistent with the output voltage profiles (Figure 5) as well as with the CV curves (Figure 6). The maximum power density reached 3.4 ± 0.3 W m^−2^ for the untreated CF, surpassing the outputs from acid-heat treated or CNF-coated counterparts. This power density value obtained in single-bottle MFCs was relatively higher than those reported in most published works, which were mostly based on two-chamber MFCs, as summarized in Table 2. In addition, the proposed simple MFCs were found to be inexpensive thanks to the absence of introducing modifying agents. Thus, this type of MFC could be of great interest for large-scale applications.

## 4. Conclusions

This comprehensive study delved into the electrochemical properties and performance of CF anodes, both in their pristine form and when subjected to various modifications. The modifications included acid-heat treatments and coatings with CNF using distinct dispersing agents. Surface morphology assessments revealed that the pristine CF anode maintained a unique 3D macroporous structure, which was further enhanced in terms of roughness upon CNF coating. Electrochemically, the pristine CF anode consistently outperformed its modified counterparts, showcasing superior electron transfer capabilities and biofilm formation kinetics. The electron transfer mechanisms within the biofilms, as evidenced using cyclic voltammetry, remained largely consistent across all anode types. However, the pristine CF anode demonstrated the most pronounced redox peaks, indicating its superior affinity for electroactive bacteria and its potential for the efficient bio-electro-conversion of substrates. Furthermore, the MFC equipped with the pristine CF anode achieved the highest power density output of 3.4 W m^−2^ with an open-circuit potential of 550 mV, surpassing the performance of those equipped with modified/treated anodes. Thus, it appears that while modifications to CF can enhance electrochemical properties, the pristine CF anode’s inherent characteristics make it a great candidate for MFC applications. Its cost-effectiveness, coupled with its electrochemical performance, underscores its potential for large-scale applications in the realm of bioenergy production. Future studies could delve deeper into the microbial communities interacting with these anodes, potentially using metagenomic and metaproteomic approaches, to further elucidate the mechanisms driving their performance. In light of the observed performance of pristine CF anodes in comparison to their treated counterparts, future research could also explore strategies to enhance the hydrophobicity of CF anodes. Increasing hydrophobicity could potentially improve electron transfer efficiency and biofilm adhesion, leading to higher power outputs in MFCs.

## Figures and Tables

**Table 1:** Summary of the performance of MFCs equipped with different anodes based on maximum power and current densities.

	CF	A-CF	CNF-SDBS@CF	CNF-SDBS@A-CF	CNF-CS@CF	CNF-CS@A-CF
OCP (mV)	−520	−540	−530	−535	−520	−520
Power density (Pmax) ± SD (W m^−2^)	3.4 ± 0.3	2.9 ± 0.2	2.7 ± 0.2	2.6 ± 0.2	2.5 ± 0.2	2.4 ± 0.2
Current density (Jmax) ± SD (A m^−2^)	26.2 ± 2.3	21 ± 1.8	17.4 ± 1.6	19.8 ± 1.8	16.5 ± 1.5	16.2 ± 1.4

**Table 2:** Comparative study of the performance of the developed MFC equipped with a pristine CF anode with those of recently reported MFCs based on unmodified and modified anodes.

No	Anode	Modification	MFC Configuration	OCP (mV)	J_SC_ (A m^−2^)	Pmax (W m^−2^)	Ref.
1	Carbon felt	PANI/m-WO_3_ ^a^	Double-chamber MFC	586	3.7	0.980	[36]
2	Carbon felt	NiO@PANI ^b^	Double-chamber MFC	725	1.5	1.078	[37]
3	NCNT ^c^/sponge	CS-NCNT-PANI ^d^	Double-chamber MFC	779	6.6	1.891	[27]
4	Carbon felt	GMC ^e^	Double-chamber MFC	800	0.3	0.070	[38]
5	Carbon felt	Br-GO f	Double-chamber MFC	630	1.0	0.240	[39]
6	Carbon felt	P/MC ^g^	Double-chamber MFC	850	4.4	1.267	[11]
7	Carbon felt	MnCo_2_O_4_ ^h^	Double-chamber MFC	780	3.5	0.945	[40]
8	Carbon felt	NiFe_2_O_4_/MXene ^i^	Double-chamber MFC	925	3.5	1.385	[41]
9	Graphite felt	PEDOT ^j^	Double-chamber MFC	1460	3.8	0.003	[42]
10	Graphite felt	Ppy-NP ^k^	Single-chamber MFC	842	6.8	1.220	[43]
		PTh-NP ^l^		644	2.2	0.800	
11	Pristine plain graphite fiber brush		Double-chamber MFC	760	7.6	2.35	[8]
12	Pristine carbon felt		Double-chamber MFC	760	3.6	1.46	[8]
13	Pristine carbon felt		Single-chamber MFC	550	26.2	3.4	**This work**

^a^ Polyaniline/mesoporous tungsten trioxide; ^b^ Polyaniline embedded in petaline NiO; ^c^ Nitrogen-doped carbon nanotubes; ^d^ Chitosan-NCNT-polyaniline; ^e^ Graphitized mesoporous carbon; ^f^ Bio-reduced graphene oxide; ^g^ Copyrolyzed pyrite and microalgae; ^h^ Manganese cobalt oxide; ^i^ Nickel ferrite/MXene; ^j^ Poly(3,4-ethylene dioxythiophene); ^k^ Polypyrrole nanoparticles; ^l^ Polythiophene nanoparticles. J_SC_ indicates maximum short-circuit current density.

## Data Availability

The data presented in this study are available in Supplementary Materials.

## Supplementary Materials

The following supporting information can be downloaded at: https://www.mdpi.com/article/10.3390/mi14122142/s1, Figure S1: MFC bottle with an air cathode; Figure S2: Silver wires used to prepare Ag/AgCl electrodes; Figure S3: Cooling the hydrogel-plugged glass Pasteur pipettes to prepare Ag/AgCl electrodes; Figure S4: Pictures illustrating the components used for single-chamber MFC experimentation and setup; Figure S5: The conventional three-electrode electrochemical cell.
