# Supplementary material for: Untreated vs. Treated Carbon Felt Anodes: Impacts on Power Generation in Microbial Fuel Cells
Source: Micromachines (Basel). 2023 Nov 23;14(12):2142. doi: 10.3390/mi14122142 (PMC10744851; doi:10.3390/mi14122142)
Supplement: Supplementary file 1 [file micromachines-14-02142-s001.zip › micromachines-2699592-supplementary.pdf]

micromachines

MDPI

Supplementary Materials

Untreated vs. Treated Carbon Felt Anodes: Impacts on Power Generation in Microbial Fuel Cells

Abdelghani Ghanam [1,2], Sebastien Cecillon [1], Andrei Sabac [1], Hasna Mohammadi [2], Aziz Amine [2], François Buret [1] and Naoufel Haddour [1,*]

[1] Univ Lyon, Ecole Centrale de Lyon, INSA Lyon, Université Claude Bernard Lyon 1, CNRS, Ampère, UMR5005, 69130 Ecully, France; francois.buret@ec-lyon.fr
[2] Chemical Analysis and Biosensors Group, Laboratory of Process Engineering and Environment, Faculty of Science and Techniques, Hassan II University of Casablanca, B.P 146, Mohammedia 20000, Morocco; a.amine@univh2m.ac.ma
* Correspondence: naoufel.haddour@ec-lyon.fr; Tel.: +33-4-72-18-61-12

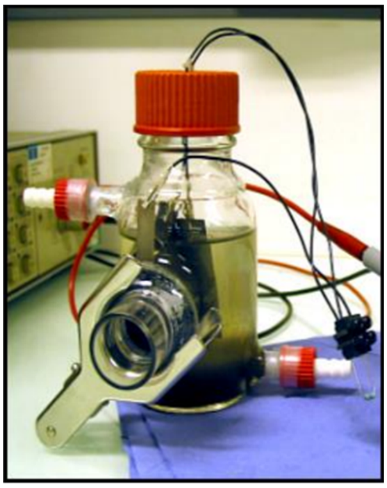

Figure S1. MFC bottle with an air cathode.

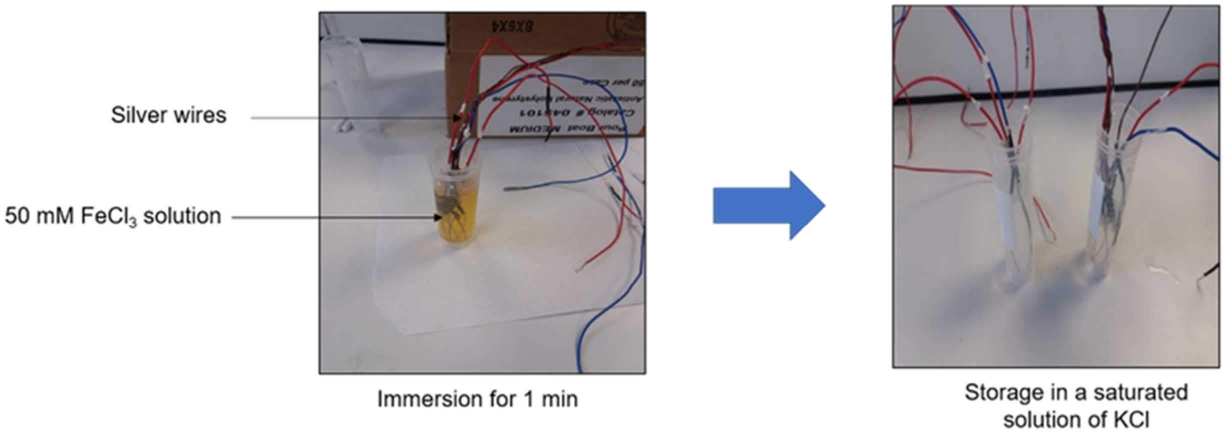

Figure S2. Silver wires used to prepare Ag/AgCl electrodes by immersing them in a solution of 50 mM $FeCl_3{\cdot}6H_2O$ (A) to form AgCl films on the silver wires, storage in a saturated solution of KCL (B).

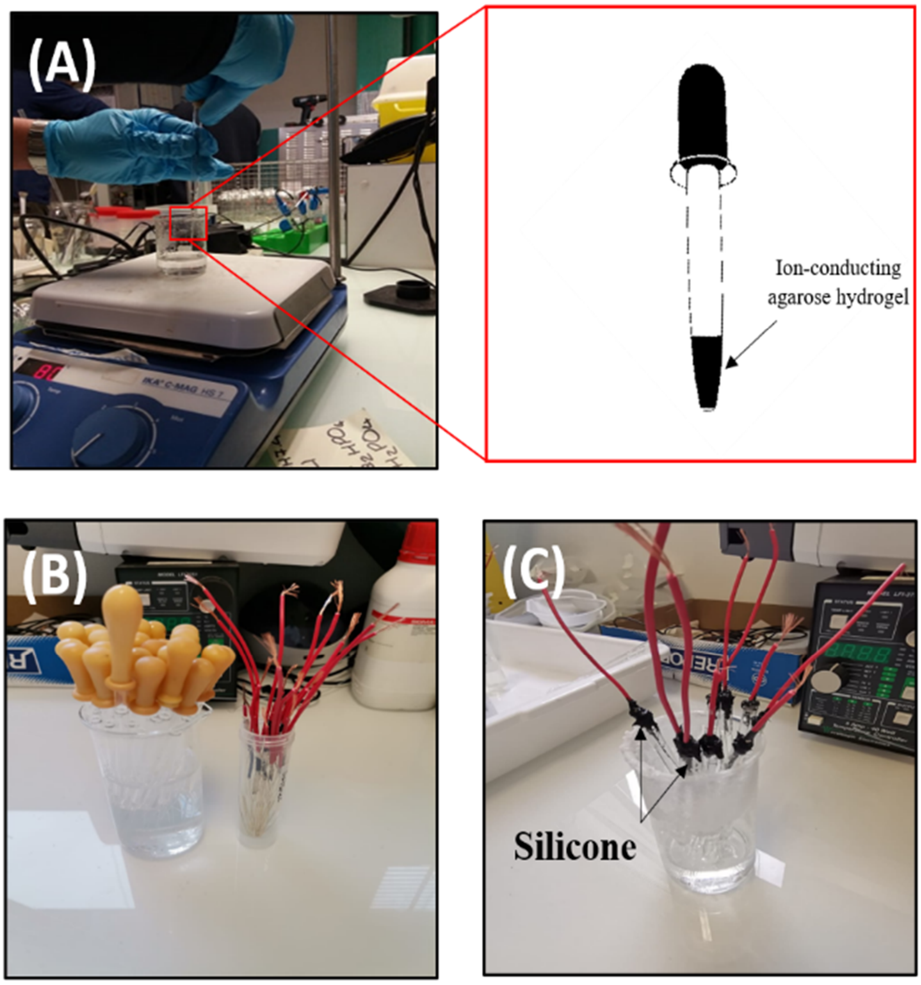

Figure S3. (A) Glass Pasteur pipettes plugging with ion-conducting agarose hydrogel. (B) Cool the hydrogel-plugged glass Pasteur pipettes at one end by immersing them in a cold KCl solution. (C) Pasteur pipette closure with silicone and stored in saturated KCl solution.

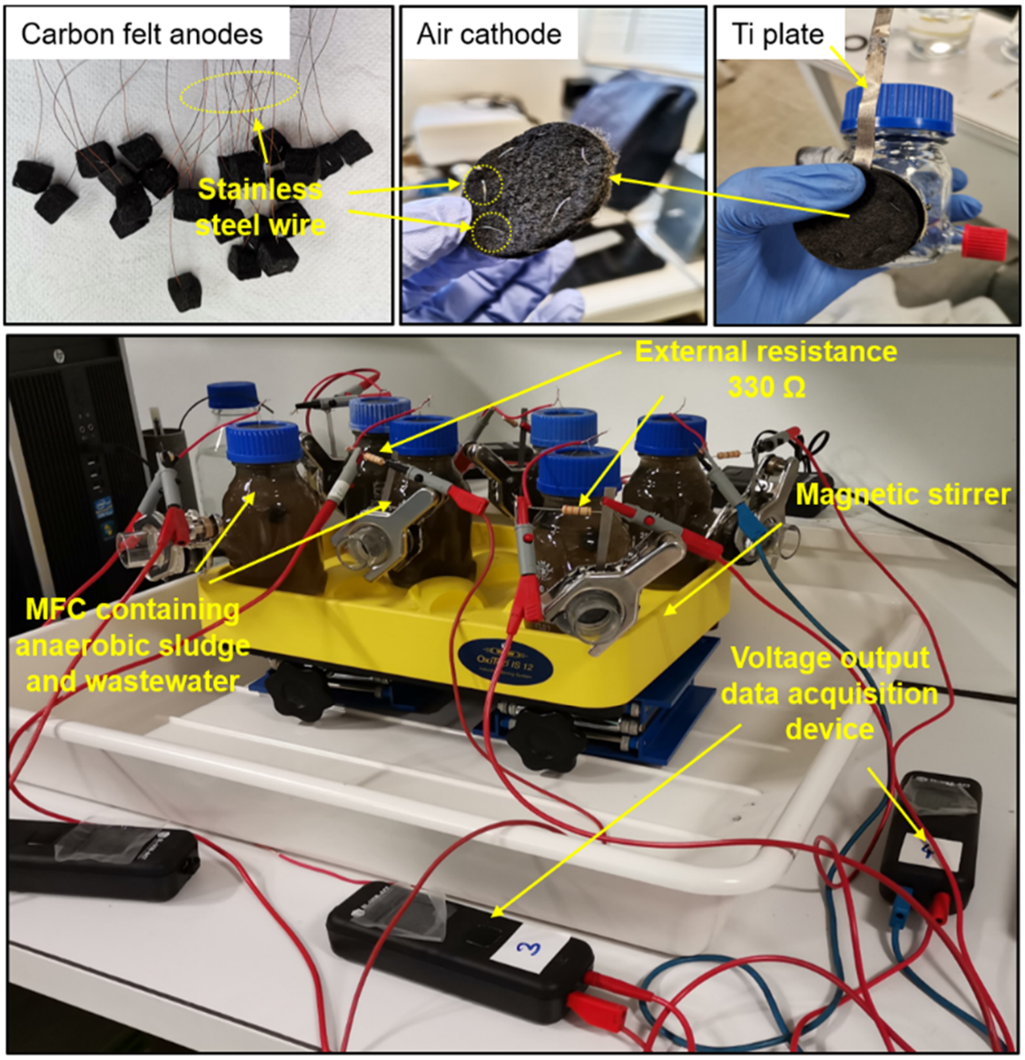

Figure S4. Pictures illustrating the components (unmodified/modified CF anodes, CF air-breathing cathodes, etc.) used for single-chamber MFC experimentation and setup. The MFC configuration used is a single-chamber bottle configuration.

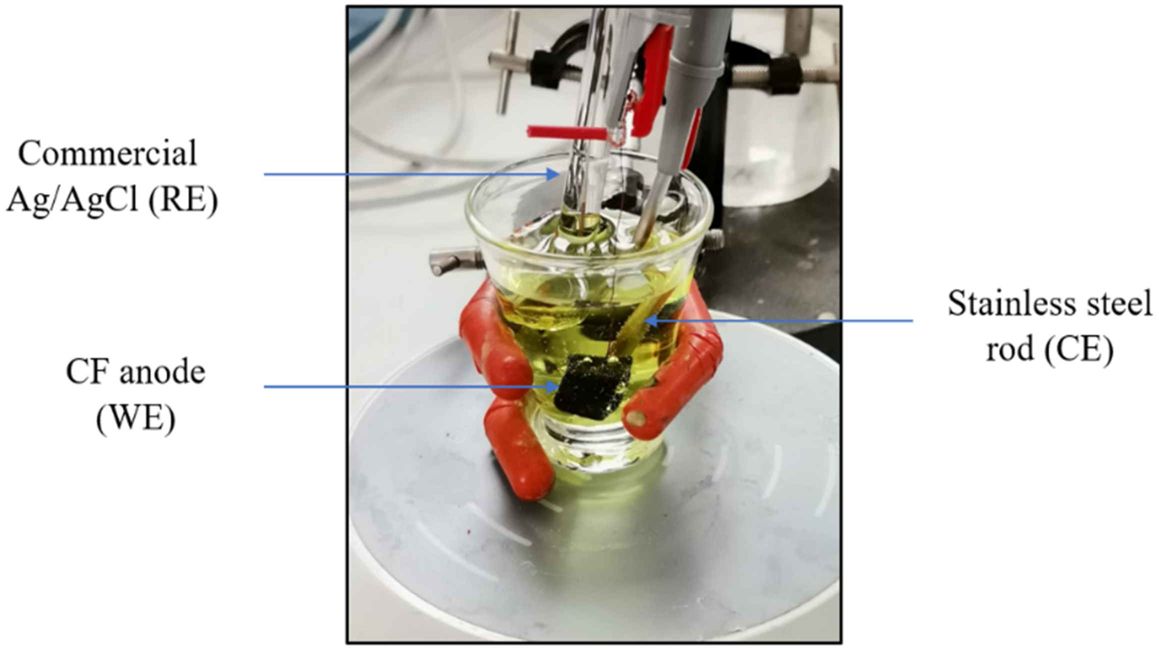

Figure S5. The conventional three-electrode electrochemical cell containing 10 mM $[Fe(CN)_6]^{3-/4-}$ dissolved in 0.1M KCl.
